# Priorities for an African research agenda on climate, environment and maternal, newborn and child health

**DOI:** 10.3389/fgwh.2026.1814330

**Published:** 2026-08-18

**Authors:** Prestige Tatenda Makanga, Tamara Rosemary Govindasamy, Sibusisiwe Audrey Makhanya

**Affiliations:** 1 Place Alert Labs, Surveying and Geomatics Department, Faculty of the Built Environment, Midlands State University, Gweru, Zimbabwe; 2 Climate Environment and Health Department, Centre for Sexual Health and HIV AIDS Research, Harare, Zimbabwe; 3 Department of International Public Health, Liverpool School of Tropical Medicine, Liverpool, United Kingdom; 4 IBM Research, Johannesburg, South Africa

**Keywords:** African context, air pollution, climate, environment, health impact, maternal and child health

## Abstract

Climate change and environmental degradation are reshaping maternal, newborn and child health risks across Africa, yet evidence generation remains constrained by sparse monitoring infrastructure, fragmented health and environmental data, limited data linkage, and uneven governance capacity. This Perspective proposes priorities for an African research agenda on climate, environment and maternal, newborn and child health. It argues for research that is continentally relevant but locally grounded: designed for data scarcity, attentive to climate and environmental heterogeneity, reflexive about data sovereignty, and embedded in translation and capacity strengthening. Drawing on experience from the CHEAQI-MNCH project and related work in selected West, East and Southern African settings, we outline seven priorities for African-led, context-sensitive and action-oriented climate and environmental health research.

## Background: climate, environment, and health in Africa

Africa is experiencing some of the most rapid and complex changes in climate, the environment, and population health. Worsening air quality, rising temperatures and associated intensifying climate extremes are leading to more frequent and intense landscape fires, flooding, droughts, and rapid land-use change, reshaping environmental exposure patterns across the continent (1). These environmental stressors intersect with rapid urbanisation characterised by informal settlement growth, persistent energy poverty, and constrained health systems. For the continent, this is leading to increases in multiple health risks with the resultant increase in the burden of diseases which further constrains already weakened health systems.

Pregnant women, newborns, and children are particularly sensitive to changes in the environment. Adverse environmental exposures during pregnancy and early life, coincide with periods of heightened biological sensitivity that is further mediated by deficiencies in nutrition, access to care, and other socioeconomic conditions (2). Heat exposure has been associated with preterm birth, low birth weight and stillbirth; air pollution and landscape fire smoke may affect fetal growth and child respiratory health; flooding and drought can disrupt antenatal care (3), delivery care, referral pathways, nutrition, water quality and infectious disease transmission; and food insecurity, displacement and livelihood loss may worsen maternal and child vulnerability (4). These pathways are shaped by nutrition, housing, occupation, access to water, access to care, household energy use and broader socioeconomic conditions. Yet despite this vulnerability, evidence from African settings remains limited, fragmented and often poorly integrated into global climate-health assessments.

A central constraint to quantifying environmental impact on health is the persistent scarcity and uneven distribution of environmental and weather monitoring infrastructure. For example, large parts of the continent lack routine ground-based air quality monitoring, high-resolution climate observations, or integrated environmental health information systems (5). At the same time, there are growing volumes of underused data for Africa, including satellite products, reanalysis datasets, surveys, and personal, low-cost environmental sensor data. Therefore, for Africa, it can be argued that there is not only a routine monitoring data scarcity problem, but also one of ineffective reuse, failed linkages, and poor governance of existing alternate datasets that are relevant in assessing environmental exposures.

The growth and strengthening of the health research community in Africa has spurred the growth in the collection of cohort, clinical trial, electronic health records and demographic and health survey datasets. However, due to the sensitive nature of health data, access remains a challenge. This creates a paradox. On one hand, legitimate concerns around data privacy, sovereignty, and regulatory compliance (particularly for health and geolocated data) can constrain data sharing and reuse. On the other, if governance frameworks are thoughtfully designed, these same constraints can promote trust, standardisation, and more efficient knowledge production. Navigating this tension is central to advancing climate and environmental health research in Africa.

This article is a Perspective rather than a systematic review or empirical multi-country analysis. It is informed by the authors' experience in the CHEAQI-MNCH (Characterising Effects of Air Quality In Maternal, Neonatal and Child Health) (6) project and related climate, environment and MNCH work across selected sub-Saharan African settings, including countries in West, East and Southern Africa. These settings do not constitute a representative sample of the continent, and the paper does not claim empirical generalisability in the manner of a formal comparative study. Rather, we use this experience reflexively, alongside selected peer-reviewed literature and regional policy processes, to identify recurring methodological, governance and translational challenges that resonate with wider African climate-health priorities. The intention is to offer an agenda-setting Perspective: grounded in specific African research experience, aligned with continental policy concerns, and intended for adaptation to diverse national and subnational contexts.

## Priorities for climate and environmental health research in Africa

The background above highlights linked challenges central to climate and environmental health research in Africa. These challenges suggest that African climate and health research must move beyond documenting risk to generating credible evidence under data scarcity, responsibly linking fragmented datasets, and designing research that supports policy, implementation, and health-system resilience. Drawing on experience and lessons from the CHEAQI-MNCH Project (Characterising Effects of Air Quality In Maternal, Neonatal and Child Health) (6) which investigates impact of air quality on maternal, neonatal and child health in African settings, the authors propose the stated points in the rest of the article as practical areas for strengthening African-led, context-sensitive, and action-oriented climate and environmental health research.

## Designing research for structural data scarcity

Reliable and accessible environmental exposure and climate data (including air quality) remains a critical limitation for informed decision-making, targeted interventions and policy contributions in public health across Africa (7). Addressing opportunities to fully utilize the geographically constrained network of air quality monitoring stations, is imperative for advancing robust research frameworks. Climate and environmental health research in Africa must be designed to function under conditions of persistent data constraints. Sparse ground monitoring is widely recognised as a structural reality rather than a temporary gap. As such, the central target should not necessarily lean towards immediately achieving ideal measurement at scale as this will take time and is capital intensive. Focus should be on generating credible, transparent, and decision-relevant exposure information using these existing data sources.

This priority supports the use of proxy indicators, satellite-derived products, modelled exposure surfaces, and hybrid data strategies that combine multiple imperfect sources. It is crucial to highlight the importance of validation, explicit uncertainty characterisation and clarity about appropriate use of proxy or derived data products within African contexts. Exposure assessment should be framed as a tool for inference and action, rather than an end in itself or a replication of regulatory paradigms developed elsewhere on the globe (8).

## Accelerating data reuse through context-appropriate governance

Across African settings, we recognise the untapped potential of existing environmental and health data. Satellite archives, reanalysis products, cohort datasets, health facility records, and survey data offer opportunities to accelerate evidence generation without duplicating data collection. However, the concerns about how data privacy, consent, geolocation, and cross-border data sharing (shaped by emerging national legislation, institutional policies, and international regulations) often limit reuse.

Rather than viewing these constraints solely as barriers, there is need for proportionate, context-aware data governance frameworks. Approaches such as trans-boundary data sharing policies and employing data curation strategies that enable data linking to facilitate reuse while protecting individual and community interests are essential (9). For climate and MNCH data, responsible reuse also requires attention to data ownership, custodianship, sovereignty, benefit-sharing and African institutional leadership, particularly when geolocated health data are linked with environmental exposures. When well designed, such frameworks can enhance trust, reduce duplication, and speed up cumulative production and translation of knowledge particularly in multi-country African research collaborations.

## Conceptualising exposure as socio-environmental and multi-source

Environmental exposures typically have both physical and social system origins. For example, outdoor and indoor air pollution and household energy use, waste burning and landscape fires, land cover and land use change are influenced by meteorology, settlement and mobility patterns. Narrow, single-source conceptualisations of environmental exposure (e.g., heat or air quality alone) prove to be insufficient for MNCH, where vulnerability is shaped by both biological sensitivity and social context. Systems oriented exposure assessments, based on integrated socio-environmental frameworks which capture cumulative risk, co-exposures, and social mediation, better reflect lived experience and challenge hazard centric approaches. This underscores the need for integrated socio-environmental frameworks that capture cumulative risk, co-exposures, and social mediation (10).

## Prioritising temporal and climate variability

Seasonal fire regimes, episodic pollution spikes, heatwaves, and rainfall variability shape exposure patterns in ways that static averages fail to capture. The temporal nature of climate and environmental exposures in Africa is a crucial consideration. Annual or long-term exposure averages obscure the short-term dynamics most relevant for health, particularly during pregnancy and early childhood. This aligns with growing evidence that acute exposures and climate extremes have disproportionate impacts on birth outcomes and child health (2, 11). Temporally resolved exposure assessments that are capable of capturing lags, cumulative effects, and periods of heightened sensitivity, should therefore be treated essential for climate health research in African settings.

## Local context is a foundation for credibility and uptake

Differences in climate, urban form, infrastructure, energy systems, and governance shape both exposure patterns and health outcomes, while exposure-health relationships vary substantially across African regions and climatic zones. There should be strong caution against naive transfer of models, assumptions, meaning or thresholds developed in other regions.

Scientific credibility and policy uptake should be seen as contingent on local validation, contextual grounding, and African leadership in model development and interpretation. Even when global datasets are used, their application must be interrogated and adapted to local and personal realities if findings are to be trusted and acted upon (12).

## Valuing methodological realism and transparency

Rather than prioritising unattainable precision, researchers should embrace approaches that clearly articulate assumptions, limitations, and uncertainty—following a pragmatic orientation toward technical methodologies. Potential mischaracterization of exposure due to poor data infrastructures should not only be perceived as a limitation. Pragmatic exposure assignment strategies could be acceptable where ideal data are unavailable, provided their implications are transparently communicated.

Researchers should also be increasingly open to advanced analytical methods, including machine learning, where these help capture non-linear and context-dependent relationships, and where conventional statistical approaches fail due to data inadequacies. Such approaches are valuable, not only for novelty, but for their capacity to improve inference and hypothesis generation under complexity (13).

## Embedding translation and capacity strengthening

Climate and environmental health research in Africa should inform action. Evidence generation is closely linked to health system planning, environmental management, policy development, and early warning systems. Translation to practice should therefore be embedded within research design, not treated as a downstream add-on (14). Equally, capacity strengthening, in the form of skills development, institutional support, and cross-sector collaboration, is inseparable from research impact. Strengthening African research ecosystems should be a prerequisite for sustained climate health resilience (15).

## Discussion: implications for African climate–health research agendas

Taken together, these priorities point to a climate and environmental health research agenda in Africa that is pragmatic, systems-oriented, and intentionally translational. They challenge deficit narratives that frame African contexts primarily in terms of what is missing and instead foreground strategies for generating credible evidence under constraints and complex realities of the African context. The argument is therefore continental in ambition but context-sensitive in application: it identifies shared structural constraints while recognising that priorities must be adapted to national and subnational realities.

Importantly, they also highlight the strategic importance of data reuse and governance. As climate risks intensify, accelerating knowledge production will depend less on generating entirely new data and more on responsibly reusing and linking existing data. Privacy and regulatory frameworks, if proportionate and context appropriate, can serve as enablers rather than obstacles, supporting trust, collaboration, and cumulative science.

Aligning research agendas with these priorities requires shifts in how excellence is defined. Funders, journals, and institutions must recognise context-sensitive, governance-aware, and translational research as markers of rigor rather than compromise. Without such alignment, climate health research risks producing evidence that is scientifically sophisticated but operationally disconnected from African realities.

## Conclusion

Africa faces escalating climate and environmental health risks, with disproportionate impacts on women, newborns, and children. Priorities articulated here emphasise the need for scientifically valid research which embeds translation and capacity strengthening at its core and is conducted to overcome the challenges of data scarcity and to accelerate responsible data reuse. The intention must be to capture or account for socio-environmental complexity, spatial and temporal dynamics.

While grounded in the authors perspectives emerging from work on air pollution and MNCH, these insights could be broadly applicable across climate-sensitive health outcomes. They offer a framework for rethinking climate and environmental health research in Africa, one that prioritises relevance, equity, and impact alongside scientific rigor.

## Data Availability

The original contributions presented in the study are included in the article/Supplementary Material, further inquiries can be directed to the corresponding author/s.
